# External sources promoting resilience in adults with intellectual disabilities: A systematic literature review

**DOI:** 10.1177/1744629520961942

**Published:** 2020-09-28

**Authors:** Femke Scheffers, Xavier Moonen, Eveline van Vugt

**Affiliations:** MEE ZHN, The Netherlands; 1234 University of Amsterdam, The Netherlands; 1234 University of Amsterdam, The Netherlands; 1234 University of Amsterdam, The Netherlands

**Keywords:** intellectual disability, resilience, adversity, support network

## Abstract

**Background:**

Persons with an intellectual disability are at increased risk of experiencing adversities. The current study aims at providing an overview of the research on how resilience in adults with intellectual disabilities, in the face of adversity, is supported by sources in their social network.

**Method:**

A literature review was conducted in the databases Psycinfo and Web of Science. To evaluate the quality of the included studies, the Mixed Method Appraisal Tool (MMAT) was used.

**Results:**

The themes: “*positive emotions*,” “*network acceptance*,” “*sense of coherence*” and “*network support*,” were identified as sources of resilience in the social network of the adults with intellectual disabilities.

**Conclusion:**

The current review showed that research addressing sources of resilience among persons with intellectual disabilities is scarce. In this first overview, four sources of resilience in the social network of people with intellectual disabilities were identified that interact and possibly strengthen each other.

There is considerable agreement about the detrimental effects of adverse life events (Vervoort-Schel et al., 2018). Both short and long term mental and physical health problems have been identified, such as depression, anxiety, and risky behaviors which could result in an increased use of health care services (Beards et al., 2013; Bethell et al., 2014; Kalmakis and Chandler, 2015; Michl et al., 2013; Tolin et al., 2010). People with an intellectual disability are at increased risk of experiencing adversity throughout their course of their life (Vervoort-Schel et al., 2018: Wigham et al., 2011; Wigham and Emerson, 2015). In Hastings et al.'s (2004) study, adverse life events were measured in people with intellectual disabilities. Almost half of the research population (46.8%) had experienced one adverse life event in the past year, while 17.4% experienced two or more adverse life events.

Research shows that the prevalence of adverse life events is higher for people with intellectual disabilities compared to the general population (Hulbert-Williams and Hastings, 2008). For instance, prevalence rate studies show that people with intellectual disabilities are at a higher risk of experiencing sexual abuse, with prevalence rates varying between 7 and 34 percent (Byrne, 2018; Gil-Llario et al., 2019; Lin et al., 2009; Mitra et al., 2011). In contrast, prevalence rates in the general population vary from 4 to 21.4 percent (Chen et al., 2010). A possible explanation for the increased risk for adversity in people with intellectual disabilities is that a number of characteristics which are associated with resilience, among which cognitive skills, executive functioning, self-efficacy, economical security, and close relationships to peers, family and mentors, are often limited or under stress in people with intellectual disabilities (Burt and Paysnick, 2012).

Research on successful aging in older adults with intellectual disabilities is very scarce (Coppus, 2013). Improvements in health care are leading to a greater life expectancy of people with intellectual disabilities (Bigby, 2002; Dew et al., 2006; Tyrer et al., 2007). As the life expectancy of people with intellectual disabilities is increasing, research should focus specifically on how to promote well-being in adulthood. Shogren (2013) reviewed articles published in the field of positive psychology to determine the degree to which disability (in general, not specific to intellectual disability) was represented in that literature base. Six (4%) of the 162 articles of The Journal of Positive Psychology explicitly mentioned people with disabilities. A similar search, but specifically on resilience in adults with intellectual disabilities, was performed by Scheffers et al. (2020). In a first search for studies on resilience in people with intellectual disabilities only six such studies were identified (Scheffers et al., 2020). Masten et al. (2002) showed that research on resilience started in the 1960s. However, research on resilience in people with intellectual disabilities can only be found from 2006 onward (Scheffers et al., 2020). Since research has established a large body of evidence on the risk factors in adults with intellectual disabilities, the time has now come to focus on the resilient characteristics. In the current study, a framework is provided of the available research on sources of resilience in the social network of people with intellectual disabilities.

Many variations of the definition of resilience have emerged over the years (Davydov et al., 2010; Fletcher and Sarkar, 2013). Windle (2011) performed a review on the conceptualization of resilience and constructed the following definition: “*The process of effective negotiating, adapting to or managing significant sources of stress and trauma through assets and resources.*” The three core concepts which are found among most definitions of resilience are: 1) the occurrence of adversity, 2) the presence of assets and resources to counter the effects of adversity and 3) the positive adaptation to or avoidance of a negative outcome (Windle, 2011). Following these core concepts, it is hypothesized that the occurrence of adversity is necessary for the emergence of resilience. Avoiding adversity is impossible and even unwanted since in normal development some degree of manageable stressful experiences is needed for a person to learn new life skills and become a stronger person (Aschbacher et al., 2013; Simmons and Nelson, 2007). From this perspective, the occurrence of (a manageable dose of) adversity could create opportunities to learn and gain experience.

Assets and resources can reinforce the process of resilience (Windle, 2011). Assets refer to internal sources of resilience, which are positive factors and characteristics within a person such as optimism. External sources of resilience are provided in the social network of a person. In a previous review by Scheffers et al. (2020), it was found that interaction between both internal and external sources of resilience was found in people with intellectual disabilities. Scheffers et al. (2020) noted three internal sources of resilience: *autonomy, self-acceptance and physical health.* “*A supportive social network*” was identified as an external source that could potentially facilitate the positive effects of the individual resources in a person. As an example: when parents involve the person with intellectual disabilities in decision making, this can reinforce the individual’s sense of *autonomy* and *self-acceptance,* resulting in more resilience when confronted with adverse life events. A second external resource found was “*daily activities*.” Daily activities can stimulate among others *physical activity* and successively lead to better health outcomes. Daily activities can also provide new social connections, meeting (new) friends at work or during leisure activities. To conclude, external sources of resilience were found to be able to facilitate internal sources of resilience.

Ungar (2011) states that the more a child is exposed to adversity, the more it becomes dependent on the environment for resilience. For people with intellectual disabilities, this may be especially true since they generally depend more on their social network (Bigby, 2008; Guralnick, 2006). However, the social network of adults with intellectual disabilities is often found to be much smaller compared to adults in the general population (Forrester-Jones et al., 2006; Jahoda and Pownall, 2014; Verdonschot et al., 2009). Besides this, they also experience problems with the maintenance of supportive relationships as this requires skillful social emotional functioning which is generally underdeveloped in people with intellectual disabilities (Alloway, 2010; Nord et al., 2013). While it is shown in the general population that sources of resilience can be found in the social network, this is much less often the case in people with intellectual disabilities, as their external sources for building resilience are more limited (Masten, 2018; Scheffers et al., 2020; Ungar, 2011).

Since people with intellectual disabilities are at a higher risk of experiencing adversity, and the social network plays an important role in developing resilience, more insight is necessary on the characteristics provided by the social network that can promote resilience in persons with intellectual disabilities. To date, there is no overview of research available regarding factors in the social network that can enhance resilience in people with intellectual disabilities who are faced with adversity. The research question for this study was: “*What is known in research about factors in the social network that can enhance resilience in people with intellectual disabilities who are faced with adversity?.*”

## Method

The aim of the present review was to identify factors in the social network that can enhance resilience in people with intellectual disabilities who are faced with adversity and is in line with a previously conducted literature review on resilience from the perspective of adults with intellectual disabilities (Scheffers et al., 2020). Different stages were followed in conducting this systematic literature review (Clarke, 2001; Harden and Thomas, 2005). First, a comprehensive search was performed in the databases of Psycinfo and Web of Science. To be included in the current systematic literature review different inclusion and exclusion criteria were used. The study needed to be executed in the personal and/or professional network of adults with all levels of severity of intellectual disabilities. Since the concept of resilience was the main focus of the study, when a definition of resilience was missing, the study was excluded. The included studies needed to be published in the English language. Full text had to be available to be included in the current review. Editorials were not included. Finally, studies focusing solely on the perspectives of persons in the social network and not (also) on the perspectives of the adult with intellectual disabilities were excluded.

For assessing intellectual disability, the following search terms were used: intellectual development disorder* OR mental retard* OR mental* deficien* OR slow learner* OR general learning disabilit* OR intellectual* disab*. These search terms were combined for both databases with: AND resilien* NOT (child* OR parent* OR adolesc* OR youth OR young OR teen*). Since resilience is a relatively new concept in psychology and has only been used since the 1960s, we have only searched for studies and manuscripts that were published in the period between 1960 and 2019 (Masten et al., 2002). Database limitations were set on adults with intellectual disabilities (18 years and older).

Second, to analyze the different themes in the selected studies, a narrative approach was adopted (Booth et al., 2016). Step 1 included the search for abstracts. In step 2 the studies were selected for detailed reading, while in step 3 summaries were made of all studies included in the review. In step 4 recurring themes were identified from the included studies. To evaluate the quality of the studies the Mixed Method Appraisal Tool (MMAT) (Hong et al., 2018) was used to describe the methodological quality for three domains: qualitative, quantitative and mixed-method studies. Based on the number of criteria used, a percentage was given to determine the quality of the described methodology. In Table 1 an overview of the results is presented.

**Table 1.** Descriptives of all studies included in the review.

	Authors	Year	Type	MMAT	Sample size	Population	Level^a^	Research setting	Resilience^b^
1	Aldersey, Turnbull III, & Turnbull	2014	Qualitative	100%	103	Mixed (full range)c	Unknown	Family homes and (specialized) schools	2,3
2	Grant, Ramcharan, & Goward	2003	Qualitative	80%	N.A.	Family caregivers	Unknown	N.A.	1, 2, 3
3	Grant, Ramcharan, & Flynn	2007	Qualitative	100%	N.A.	N.A.	Unknown	N.A.	1, 2, 3
4	Ingham, Riley, Nevin, Evans, & Gair	2013	Quantitative	80%	37	Professional caregivers	Unknown	Inpatient services	Unknown
5	Lee & Kiemle	2015	Qualitative	100%	9	Professional caregivers	Unknown	Medium secure forensic setting	1
6	Nevill & Havercamp	2019	Quantitative	80%	97	Professional caregivers	Unknown	Day programs and residential services	1
7	Noone & Hastings	2009	Quantitative	60%	28	Professional caregivers	Moderate to Severe	Community services	Unknown
8	Søndenaa, Whittington, Lauvrud, & Nonstad	2015	Quantitative	80%	136	Professional caregivers	Unknown	Residential and community services	1
9	Wong, Fong, & Lam	2015	Quantitative	80%	36	Family caregivers	Mild to Severe	Day programs, residential and employment services	1,2

^a^Subnote: “Level” reflects the level of functioning of the participants with intellectual disabilities, ranging from borderline intellectual functioning to profound intellectual disability. ^b^Subnote: “Resilience” shows whether a conceptualization of resilience is present; 0 = a clear conceptualization of resilience is missing, 1 = people stay on the same level of functioning even after being exposed to adverse life events (resilience), 2 = recovery from adversity (recovery), and 3 = growth beyond the original level of functioning (post-traumatic growth. cSubnote: “Full range” refers to family caregivers, extended family, friends and professional caregivers.

For every study, the main aim was to understand which factors were found that can enhance resilience in people with intellectual disabilities. In the coding scheme different types of information were coded, including general study information, sample descriptors and the conceptualization of variables such as adversity and resilience. The themes related to resilience were synthesized, overlapping themes were combined or new overarching themes were established. The classification and assessment of intellectual disabilities were coded as well as the operationalization of the concept of resilience. To objectify the process of analyzing the recurring themes, two trained research assistants rated the selected studies. Interrater reliability was found to be 0.835 which can be considered as almost perfect (Sim and Wright, 2005). Differences in coding were resolved through discussion, until agreement was obtained after which data was processed for analysis.

## Results

### Search strategy

**Figure 1.**
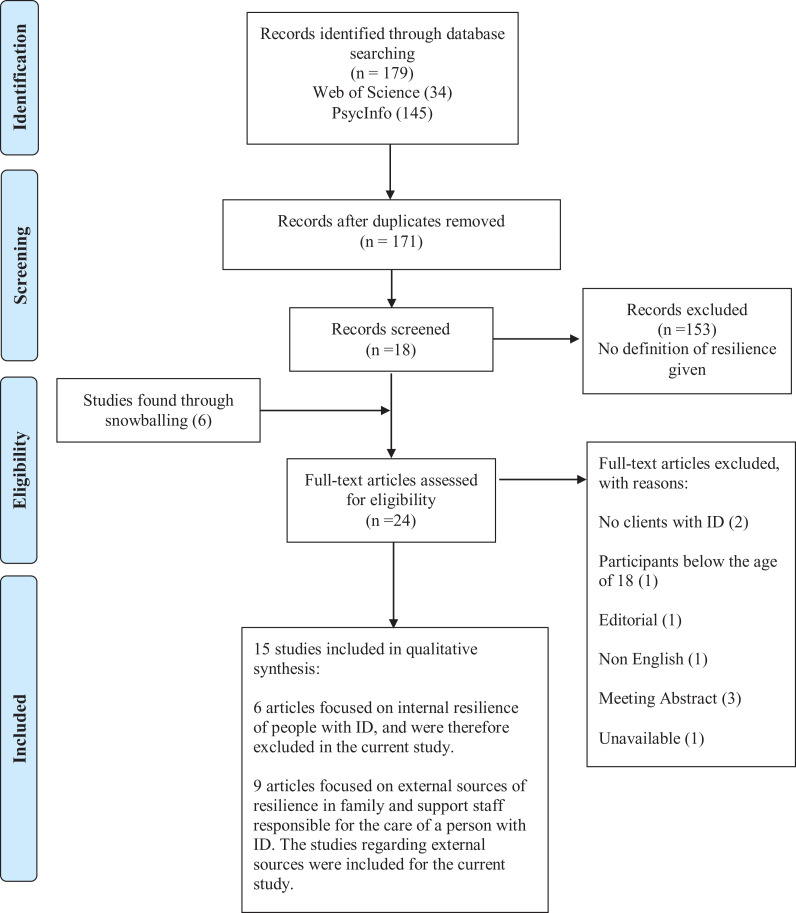
Flow chart of search strategy following PRISMA 2009 (Moher et al. 2009).

The databases Psycinfo (1960–2019) and Web of Science (1975–2019) were searched. One hundred seventy nine studies were found when combining the search terms. Eight duplicates were removed. After exclusion of studies not addressing resilience, a total of 18 studies remained. Exclusion of studies based on the inclusion criteria was done by the first author in consultation with the co-authors. Six new studies were identified through a manual search in reference lists of relevant studies. In total, 24 studies were found eligible for further inspection. After checking the titles and abstracts, nine studies were excluded after applying the inclusion criteria. Studies that did not include people with intellectual disabilities, (2) or solely addressed participants younger than 18 years of age (1) were excluded. Meeting abstracts for conferences were excluded (3). One study was an editorial note for a special issue of a journal regarding resilience in people with intellectual disabilities and was therefore also excluded. Additionally, one non-English study was excluded. One study was not available in the databases consulted. Fifteen studies remained, of which six focusing on promoting resilience on the individual level in adults with intellectual disabilities. These studies were described in another systematic literature review by Scheffers et al. (2020). The remaining nine studies uniquely focused on enhancing resilience in adults with intellectual disabilities by the social network and were therefore included in the present review. Figure 1 presents a flow chart of the search strategy following the PRISMA guidelines (Moher et al., 2009).

### Characteristics of the studies

Table 1 provides an overview of the nine studies selected. The studies were published between 2003 and 2019. Five articles had a quantitative study design and four studies had a qualitative study design. Sample sizes ranged from 9 to 136 participants. The level of intellectual disabilities was mentioned in only two studies, ranging from mild to severe intellectual disabilities, although it was not clear how this was assessed. None of the studies included people with borderline intellectual functioning. Two studies focused on family caregivers, these studies were conducted through observations in family homes (specialized schools) and interviews with parents recruited through day, residential and employment services for people with intellectual disabilities. Five studies focused on professional caregivers in different settings such as inpatient services, medium-secure forensic settings, day programs, residential and community services. In two studies a mix of family and professional caregivers were involved. Studies regarding professional caregivers showed a wide variety in professional disciplines such as persons working in a residential or ambulatory setting including residential counselors, social workers but also qualified nurses.

### Conceptualization of resilience

In the selected studies, several concepts of resilience were used: 1) people stay on the same level of functioning even after being exposed to adverse life events (resilience), 2) recovery from adversity (recovery), and 3) growth beyond the original level of functioning (post-traumatic growth) (Masten, 2018; Windle, 2011). Some studies applied more than one of these concepts, two studies discussed all three conceptualizations (Grant et al., 2003, 2007). In six out of nine studies, resilience was described as people functioning on the same level (Grant et al., 2003, 2007; Lee and Kiemle, 2015; Nevill and Havercamp, 2019; Søndenaa et al., 2015; Wong et al., 2015). The concept of recovery was applied in four studies (Aldersey et al., 2014; Grant et al., 2003, 2007; Wong et al., 2015). The concept of post-traumatic growth was discussed in three studies (Aldersey et al., 2014; Grant et al., 2003, 2007). In every study, the importance of a systemic view regarding resilience and the consideration of individual and environmental factors was mentioned.

### External sources of resilience

The data revealed different recurring themes regarding external sources of resilience. A wide variety of factors was identified that could be categorized in four themes: “*network acceptance*,” “*positive emotions*,” “*sense of coherence*” and “*network support*.” In Table 2, an overview of the different themes as found in the nine studies is presented.

**Table 2.** Overview of resiliency factors for each study.

	Authors	Positive Emotions	Network Acceptance	Sense of Coherence	Support
1	Aldersey, Turnbull, & Turnbull			X	
2	Grant, Ramcharan, & Goward	X		X	X
3	Grant, Ramcharan, & Flynn			X	X
4	Ingham, Riley, Nevin, Evans, & Gair	X	X		X
5	Lee, & Kiemle	X	X		X
6	Nevill & Havercamp		X		
7	Noone & Hastings		X		
8	Søndenaa, Whittington, Lauvrud, & Nonstad				X
9	Wong, Fong, & Lam	X		X	X

Only in studies regarding professional caregivers was *network acceptance* mentioned as necessary to facilitate resilience (Ingham et al., 2013, Lee and Kiemle, 2015; Nevill and Havercamp, 2019; Noone and Hastings, 2009). Network acceptance refers to the ability of important persons in the network to experience acceptance toward the person with intellectual disabilities regarding their qualities and limitations, thus creating an environment where problem-solving skills are optimally applied. Through an accepting coping style, professionals were able to stay calm and provide support to enforce resilience in the adult with intellectual disabilities. Without acceptance, the professional can be overwhelmed by emotions and, accordingly, make inadequate decisions. Network acceptance was reinforced when training professional caregivers to accept (the behavior of) the person with intellectual disabilities without judgment (Ingham et al., 2013, Lee and Kiemle, 2015; Nevill and Havercamp, 2019; Noone and Hastings, 2009). In the studies of Ingham et al. (2013) and Noone and Hastings (2009), the results of a workshop were presented focusing on facilitating resilience by the professional network dealing with challenging behavior in people with intellectual disabilities. In contrast to traditional cognitive behavioral therapy, the acceptance-based approach focuses on accepting unpleasant thoughts instead of changing or avoiding these cognitions. In the study of Nevill and Havercamp (2019), training for professionals was evaluated focusing on mindfulness to develop an acceptance-coping style in professional caregivers. By learning to be mindful, professionals were taught to observe behavior without judgment and to accept the situation as it is, and act in a calm and thoughtful manner. Nevill and Havercamp (2019) showed that training in mindfulness led to reductions in burnout and staff turnover and ultimately increased the quality of life in adults with intellectual disabilities receiving their support. Finally, the study of Lee and Kiemle (2015) showed that acceptance could be achieved by different, opposing, strategies. Firstly, “getting to know the person behind the label” was mentioned. Through experiencing various moments (both pleasant and unpleasant) the professional is able to view the client as a person with both positive and negative characteristics. Secondly, “remaining emotionally distant” when faced with challenging behavior was mentioned to be able to deploy an acceptance coping style. Remaining emotionally distant could prevent professionals from being overwhelmed by emotions. This enables professionals to be better able to adjust their actions to the needs of the person with intellectual disabilities.

Expressing *positive emotions* was mentioned in four out of nine studies (Grant et al., 2003; Ingham et al., 2013; Lee and Kiemle 2015; Wong et al., 2015). By stimulating positive emotional responses in professional caregivers stress levels were reduced and positive interactions with persons with intellectual disabilities were reinforced. Consequently, resilience in persons with intellectual disabilities was stimulated by focusing more on positive instead of challenging behaviors. Different suggestions were made on how positive emotions can be stimulated. It is important to be able to spend a sufficient amount of time together (Lee and Kiemle, 2015) To create opportunities to experience positive moments next to unpleasant moments. Through positive moments, the professional was able to learn about the qualities and strengths in the person with intellectual disabilities.

Positive emotions were also mentioned as an important factor in facilitating resilience in parents of people with intellectual disabilities. From the perspective of family caregiving, Grant et al. (2003) suggest that very small improvements in the behavior of people with intellectual disabilities can generate a sense of reward, leading to the development of positive emotions such as hope or optimism among caregivers. Hope and optimism result in more positive interactions thus facilitating sources of resilience for the person with intellectual disabilities. Caregiver satisfactions can be increased by professionals by mentioning the observation of improvements in the behavior of the person with intellectual disabilities to their family caregivers.

Antonovsky's (1987) theory was mentioned in two studies to explain the concept of a “*Sense of Coherence*” (Grant et al., 2003, 2007). A sense of coherence is a mixture of optimism and control and is defined by Antonovsky (1987) as: “*The extent to which one has a feeling of confidence that one’s environment is predictable and that things will work out as well as can reasonably be expected.*” Being able to maintain a sense of coherence after experiencing an adverse event can have a key impact on staying psychologically healthy (Grant et al., 2003). Making meaning of an adverse event can help to understand and take control of the event by addressing suitable resources of resilience and developing the ability to re-invent oneself to cope with future adverse events (Grant et al., 2003, 2007). *Meaningfulness, comprehensibility* and *manageability* form the core of a sense of coherence. Aspects addressing a sense of coherence were mentioned in four out of nine studies including both family and professional caregivers (Aldersey et al., 2014; Grant et al., 2003, 2007; Wong et al., 2015).

In Aldersey et al.'s (2014) study, different strategies for meaning-making were addressed. In that study, the origin of intellectual disabilities was either seen as having a biomedical, metaphysical or a combined cause. The perspective that the family adopted in dealing with intellectual disabilities has a great influence on the kind of support the person with intellectual disabilities is likely to receive. For example, a metaphysical explanation means that the family sees the intellectual disabilities as a result of sorcery, broken taboos or fetishes resulting in stigmatization from the community. This view can prevent the family from seeking and giving support to the person with intellectual disabilities, possibly even isolating the person with intellectual disabilities for fear of being shunned by the community. Families that adopt a combined perspective of a biomedical and metaphysical model are expected to engage in a more pluralistic support seeking pattern. This process will make more resources available to the family in teaching a person with intellectual disabilities to deal with adversity. In Wong et al.'s (2015) study, a different strategy was shown to reinforce the concept of “meaningfulness.” In this study parents of people with intellectual disabilities would make regular visits to older adults who lived alone. The study suggests that talking about the life stories of these older adults could help the parents to self-reflect on their own meaning of life and pass that on to their children with intellectual disabilities (Wong et al., 2015). After this program the parents experienced significant enhancements with regard to meaningfulness.

Comprehensibility is essential to offer insight regarding adverse events to the family in an understandable manner (Grant et al., 2003, 2007). In dealing with specialized services, maintaining comprehensibility and control seemed more difficult. In contact with services, procedures could sometimes come across as arbitrary or unfair. Service providers need to work in a transparent way to support a family in making meaning of a situation and gaining control. A sense of control can help parents to become active agents in supporting resilience in the child and can prevent parents from feeling “captives” of the circumstances in their life (Grant et al., 2007).

The concept of manageability means that a person has access to sufficient resources to deal with adversity. By maintaining structures and boundaries a person is able to manage a chaotic environment. Each family has its own unwritten rules and norms in dealing with everyday life, also referred to as family schema. These schemas help families to determine to what extent support is accepted and from who. Consequently through family schemas it is determined which resources for people with intellectual disabilities are available in dealing with adversity. For instance, parents going beyond “normal” borders to protect and care for their children can lead to a higher degree of tenacity. Further, it can also lead to a perspective whereby “outsiders” are viewed as incapable or inadequate in caring for the person with intellectual disabilities, thus restricting possible resources people with intellectual disabilities can rely on.

*Network support* was mentioned in six out of nine studies. By providing network support to families and professional caregivers, it was possible to teach the person with intellectual disabilities how to deal with adversity in a resilient way (Grant et al., 2003, 2007; Ingham et al., 2013; Lee and Kiemle, 2015; Søndenaa et al., 2015; Wong et al., 2015). Parents of persons with intellectual disabilities need support from their own network to experience that they are not alone (Grant et al., 2003, 2007). A common fear in parents of children with intellectual disabilities is: “What will happen to my child when I am not able to care for him/her anymore?” Through support from their own network these fears can decrease since the parents know other people will be available to care for their child. This will reduce levels of stress thus increasing opportunities to experience positive moments with their child and enhance resilience in their child. In the study of Wong et al. (2015) the volunteer program provided new opportunities to expand the social network of the family and the person with intellectual disabilities. Thus creating a platform for exchanging experiences on how to provide the best care and create support opportunities for the person with intellectual disabilities who faces adversity. The level of success of this volunteer program was enhanced by a number of debriefing sessions. These sessions provided a platform to laugh and cry together leading to more effective coping skills in other life domains as well. Finally, network support for parents also means that the parents have confidence in the available care. With confidence in the healthcare system, more resources will be made available to the person with intellectual disabilities.

People with intellectual disabilities need specific care. When organizations are able to support their professionals adequately this, in turn, will facilitate professional actions aiming at enhancing resilience in persons with intellectual disabilities. Different factors can contribute to a feeling of “being supported” for professional caregivers. A long-term perspective can be beneficial for professionals working with people with intellectual disabilities (Søndenaa et al., 2015), since trajectories in care for people with intellectual disabilities often have a lifelong character. A high continuity of staff caring for people with intellectual disabilities is very important in facilitating them to focus on resilience in persons with intellectual disabilities. In the study of Lee and Kiemle (2015), it was shown that working with a specific subpopulation is related to a specific level of tolerance. The attribution of the challenging behavior (i.e. labeling a person as a victim or a perpetrator) is important in constructing the attitude of the professional and influences the interaction between the professional and the person with an intellectual disabilities and the professional attitude to support resilience in persons with intellectual disabilities. Finally, the importance of supervision and staff support to address resilience in persons with intellectual disabilities needs to be emphasized for professionals working with people with intellectual disabilities and challenging behavior (Lee and Kiemle, 2015). The informal sharing of feelings with colleagues or a formal supervisors enables professionals to provide better support people with intellectual disabilities.

## Discussion

People with intellectual disabilities have a dependency on their environment. Therefore, their social network is a key factor in the process of facilitating resilience (Kittay, 2011). The current systematic literature review provides an overview of the available research on how to strengthen resilience in people with intellectual disabilities through their social network. It is through the social network that it is possible to unfold an individuals’ qualities and thus kickstart positive growth in dealing with adversity. In the current study, the social network included both the personal and professional network.

Practitioners and policy makers have largely focused on identifying vulnerabilities and risks among people with ID to align the right intensity of care. Through the focus on risks little is known about the strengths people with ID possess and how people with ID are able to manage risks. New developments in research are usually much later applied to the population of people with intellectual disabilities (Feldman et al., 2014; Lai et al., 2006; Mactavish et al., 2000), which also appears to be the case regarding research on resilience (Scheffers et al., 2020). In a review study by Windle et al. (2011) only 15 instruments assessing resilience were identified. None of these instruments were adapted to the needs and capacities of people with intellectual disabilities. In addition, none of the instruments explicitly included people with intellectual disabilities in the validation procedure. Consistent with the findings from the current review there appears to be a huge gap in research focusing on resilience in people with intellectual disabilities and their social network.

The current study provides a first insight in the process of resilience in people with intellectual disabilities from the perspective of the social network. These themes can be used to adapt policies or interventions to fit the resilient capacities of people with intellectual disabilities taking into account the level of functioning. The themes with regard to resilience identified in the current review are: “*positive emotions*,” “*network acceptance,*” “*sense of coherence*” and “*network support.*” Findings from the current systematic literature review suggest that these themes are not exclusive categories. Enhancing resilience is a complex and dynamic process, and no one theme is expected to uniquely contribute to resilience. Below, it is discussed how the identified themes are interconnected and build on the qualities of other themes.

*Network acceptance* referred to the ability of important persons in the network to experience acceptance toward people with intellectual disabilities, thus enhancing resilience in people with intellectual disabilities. Network acceptance may, for instance, help professionals to find the right balance of expectations toward the person with intellectual disabilities. Subsequently this may limit the risk of overestimation and use of incorrect support by focusing too much on the occurrence of challenging behavior. Workshops based on mindfulness, acceptance and commitment therapy appear to be useful in enforcing an acceptance-based coping style. In a study by Lietz (2007), it was shown that acceptance is also considered an important stage in the process of family resilience, and expected to serve as a base for building resilience in a person with intellectual disabilities. An accepting coping style can be supported by humor, insight, open communication and a belief system that provides comfort (Lietz, 2007). Through network acceptance it is expected that a cornerstone is laid for “*positive emotions.*”

*Positive emotions* are widely recognized as being important resources for supporting resilience (Johnson et al., 2010; Mak et al., 2011; Ong et al., 2010). For family caregivers, it is important that small improvements in the behavior and development of the person with intellectual disabilities are recognized to enhance positive and stimulating interactions with the person with intellectual disabilities (Grant et al., 2003). Trainings or workshops for professional caregivers on reframing adverse events by positive emotions can also help to prevent burnout symptoms and high rates of staff turnover (Ingham et al., 2013; Lee and Kiemle, 2015; Noone and Hastings, 2009; Søndenaa et al., 2015). Following the broaden-and-build theory, professionals showing positive emotions can help people with intellectual disabilities to develop resources for resilience (Fredrickson, 2013). For instance, when a professional expresses more positive emotions, the social network of that person could be more motivated to support to the person with intellectual disabilities in difficult times. The concepts “*network acceptance*” and “*positive emotions*” seem to interact and strengthen each other. Through these capacities a following cornerstone is laid for a “*sense of coherence.*”

*Sense of coherence* goes beyond situational acceptance. Through a sense of coherence a person tries to understand the meaning of an event, and based on this evaluation, find appropriate resources for supporting resilience. The process of “making meaning” of adversity has a great influence on how an external threat is perceived, cognitively processed and integrated into an adaptive family schema including that of the family member with intellectual disabilities (Grant et al., 2003). Through understanding and meaning-making of adverse events caregivers develop a sense of control that they can try to transfer to the person with intellectual disabilities (Grant et al., 2003, 2007). In the study by Olsson and Hwang (2002), it was shown that parenting a child with intellectual disabilities is a constant stressor that negatively influences the development of a sense of coherence. Possibly this explains parental difficulties to pursue specific personal life goals (Olsson and Hwang, 2002), in turn hindering their supporting capacities. Through a sense of coherence the family adopts a specific strategy in providing care for the person with intellectual disabilities. Since people with intellectual disabilities are more dependent on their network for care, it is suggested that professionals should also pay attention to the process of making meaning of adversity. For the entire family including the person with intellectual disabilities, to regain a sense of control as a system. When family and professional caregivers experience a sense of coherence, they are better able to help the person with intellectual disabilities in dealing with adversity.

Finally “*network support*” for caregivers in the personal and professional network can help to promote resilience in adults with intellectual disabilities. For family caregivers it is important to have good relationships with others thus being able to express feelings and share experiences and ideas and being a good example for their child with intellectual disabilities. Continuity of staff is important in facilitating resilience in persons with intellectual disabilities. By balancing expectations with a long-term care vision, professionals feel supported in their daily work and can provide better care (Ervin et al., 2014; Goddard et al., 2008; Kittay et al., 2005; Knotter et al., 2018). Short-term care perspectives often do not fit the needs and goals of people with intellectual disabilities since more time and trust is needed to build effective relationships and benefit from supportive care.

Several limitations should be mentioned regarding the current study. A limited number of studies was found, all published between 2003 and 2019. In the included studies, no information was given about the severity of the intellectual disabilities and how this possibly influences building resilience for people with intellectual disabilities nor were the methods to assess intellectual disabilities mentioned. The variation in research settings shows that the people with ID lived in a wide variety of settings such as: with family, a residential setting or on their own receiving care in day programs or community services. Based on the research settings, it is expected that most studies focused on higher functioning adults with mild to moderate intellectual disabilities. However, this cannot be stated with certainty since seven out of nine studies did not specify the level of severity of the intellectual disability. The population of people with intellectual disabilities is diverse, with different levels of intellectual and adaptive functioning warranting different levels and means of support (Maulik et al., 2011). Unknown yet is how this influences building resilience in people with intellectual disabilities and which support strategies are necessary to support them taking into account their specific needs. For future research it is suggested to clearly specify the severity of the intellectual disability.

Regarding the quality of the studies three studies scored positively on all the criteria of quality as assessed with the MMAT as percentages ranged from 60 to 100 percent (Hong et al., 2018). For the qualitative studies only one study scored negatively on one criteria as the coherence between data sources, analysis and interpretation was unclear (Grant et al., 2003). In the studies with a quantitative design none of the studies reported on how the study accounted for possible confounders. In one study many participants dropped out during the follow-up and the study was therefore not carried out as intended (Noone and Hastings, 2009).

The concept of (enhancing) resilience in people with intellectual disabilities is a relatively new concept in care. Further research is needed to support current findings. When trying to understand the process of resilience research should pay attention to the cultural context (Ungar, 2011). Aldersey et al.'s (2014) study was performed in the community of Kinshasa (Democratic Republic of Congo). “*Broken taboos, witchcraft, sorcery and punishment from ancestors*” were mentioned as important concepts in Kinshasa for understanding disability and explaining parental and societal actions. In Wong et al.'s (2015) study, a volunteer program was presented from Hong Kong. Respect for older adults and their life-experience is eminent in Hong Kong but perhaps not in other countries around the world (North and Fiske, 2015). Lomas (2016) published a study about untranslatable words related to well-being across different countries. It appears that many different unique non-exchangeable terms are used in various countries for well-being and positive mental health. The cultural context regarding resilience should be more adequately embedded in research.

People with intellectual disabilities can experience the same qualities of bonding with professionals as in family contacts (Van Asselt-Goverts et al., 2015). However the perspective of supporting resilience by family caregivers is underrepresented and needs urgent attention in research. In the current study only two studies focused uniquely on the personal network (family) of people with intellectual disabilities (Grant et al., 2003; Wong et al., 2015). Moreover, future research should focus on the mutual collaboration between the personal and the professional network as both networks play a major role in the lives of people with intellectual disabilities (Forrester-Jones et al., 2006; Giesbers et al., 2019; Kwekkeboom et al., 2006; Van Asselt-Goverts et al., 2015).

In the current systematic literature review four factors were identified to facilitate resilience in people with intellectual disabilities through the social network: “*positive emotions*,” “*network acceptance,*” “*sense of coherence*” and “*network support.*” To conclude, more high quality research is needed to fully understand all aspects of (promoting and building) resilience in people with intellectual disabilities who are faced with adversity in order to improve their quality of life with special attention for the social and cultural context.
